# Phacocele Induced by Traumatic Blunt Injury in a 53-Year-Old Woman: A Case Report

**DOI:** 10.22336/rjo.2025.40

**Published:** 2025

**Authors:** Bleidele Sandra, Veitners Alberts, Zemitis Arturs, Vasilcenko Irina, Bagante Ieva, Laganovska Guna

**Affiliations:** 1 Department of Ophthalmology, Riga Stradins University, Riga, Latvia; 2 Pauls Stradins Clinical University Hospital, Riga, Latvia; 3 Department of Oral and Maxillofacial Surgery and Oral Medicine, Riga Stradins University, Riga, Latvia

**Keywords:** Blunt trauma, eye globe, phacocele, subconjunctival space, crystalline lens dislocation, CT = computed tomography, OCT = optical coherence tomography, BVCA = best-corrected visual acuity, IOL = intraocular lens, OTS = Ophthalmic Trauma Score

## Abstract

**Purpose:**

To report a 53-year-old woman with a traumatic phacocele after blunt eye trauma.

**Methods:**

In this case, a comprehensive ophthalmological examination, computed tomography (CT), eye ultrasonography, optical coherence tomography (OCT), as well as eye and orofacial surgeries, were performed. The surgery was divided into three different stages: a revision of the wound, maxillofacial surgery, and a 25G pars plana vitrectomy with retropupillary Artisan IOL implantation.

**Results:**

During the latest examination, occurring one and a half months after the incident, the patient presented with a best-corrected visual acuity (BCVA) of 20/70 in the left eye.

**Discussion:**

Phacocele is a rare consequence of blunt ocular trauma, most commonly observed in elderly patients after a fall, with the lens dislocated in the superonasal quadrant. Prompt surgical management is essential, although long-term visual results may be affected by postoperative complications.

**Conclusion:**

Phacocele, defined as the displacement of the crystalline lens into the subconjunctival space, is a rare complication that may arise from blunt eye trauma. Due to its infrequency, there is limited literature and documented cases available. As demonstrated in this case, the patient exhibited significant improvement in best-corrected visual acuity (BCVA) following surgical interventions in the eye.

## Introduction

Ocular trauma is a significant cause of preventable loss of vision, affecting 19 million individuals worldwide [1,2]. According to the incidence of globe injuries, there are 3.5 eye injuries per 100,000 people, with males predominating in about 80% of open globe injuries. The incidence of blunt trauma is three times less than that of sharp objects. Young people are most likely to experience blunt eye globe trauma from motor vehicle collisions, while elderly patients experience it primarily due to falls [3].

A rare condition known as phacocele is a complication of a blunt eye injury, where the crystalline lens of the eye is displaced into the subconjunctival space. Phacocele predominantly occurs in adults and is relatively uncommon in children [4,5].

## Case presentation

A 53-year-old woman was consulted in our ophthalmic emergency department due to blunt eye trauma, which occurred on February 29, 2024. The patient reported a gradual loss of vision in the left eye, accompanied by eye pain and a decrease in visual acuity. Symptoms appeared after the patient tried to chase her pet cat and fell, hitting the corner of a table with her face, resulting in blunt eye trauma. According to the patient, she had no history of eye injuries, surgeries, or diseases in the past.

### Examination

**Fig. 1:**
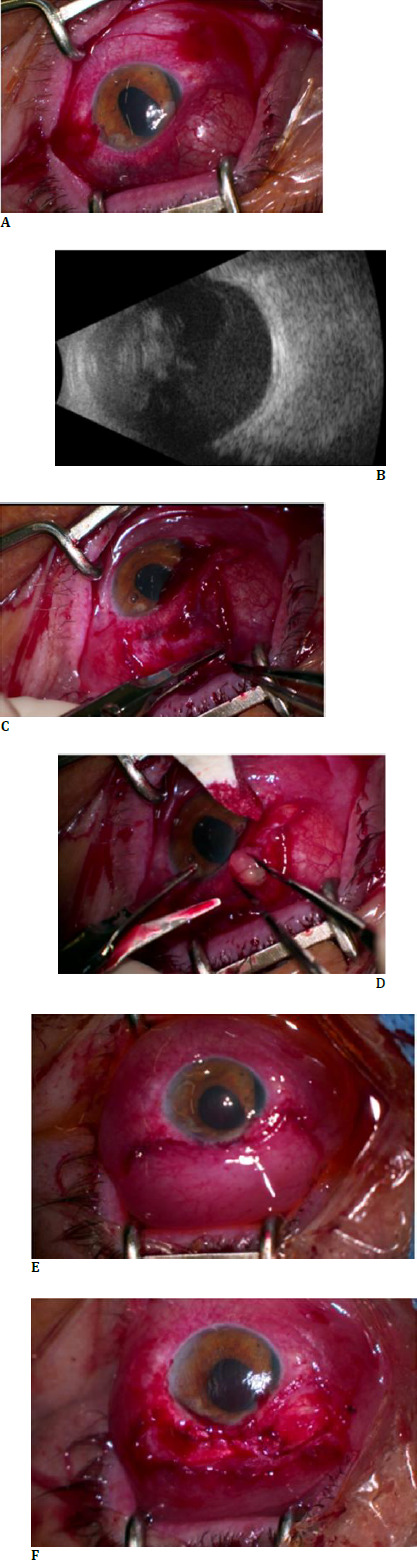
On February 29, 2024, the patient presented with diffuse conjunctival injection, nasal iridodialysis, and a dislocated crystalline lens, resulting in a bulging defect in the subconjunctival space extending from the 11 to 2 o’clock positions. The photo was captured during the initial eye operation on the day of presentation (**A**). The B-scan of the left eye showed severe hemophthalmos of the left eye on the day of presentation (**B**). The initial step of ophthalmic surgery on February 29, 2024, involved the dissection of conjunctival tissue along the 9 to 3 o’clock positions, revealing a scleral defect (**C**). The dissection of the bulging defect exposes tissues of the crystalline lens (**D**). The closure of the scleral defect using seven interrupted sutures (**E**). Repositioning of prolapsed scleroconjunctival tissues (**F**)

At presentation, her best-corrected visual acuity (BCVA) in the left eye was hand motion, and the intraocular pressure was 15 mmHg. The examination of the right eye revealed completely normal findings, with a best-corrected visual acuity (BCVA) of 1.0 and an intraocular pressure of 14 mmHg. In the left eye, diffuse conjunctival injection was found, as well as nasal iridodialysis and a dislocated crystalline lens, forming a bulging defect in the subconjunctival space along the length of the 11 to 2 o’clock positions (**Fig. 1A**). The patient presented with aphakia, and vitreous strands were observed in the anterior chamber. No conjunctival or corneal lacerations were found, and examination of the eyes’ posterior pole was complicated due to vitreous haemorrhage. A B-scan examination revealed a severe vitreous haemorrhage within an intact eye globe, with no signs of scleral or retinal breaks (**Fig. 1B**). The patient also underwent computed tomography (CT) imaging of the head and orbits, revealing left orbital and maxillary fractures accompanied by dislocations.

The patient’s surgical management was conducted in three distinct stages, involving both the ophthalmology department and the Department of Oral and Maxillofacial Surgery. Dr. Arturs Zemitis, a vitreoretinal surgeon, performed all ophthalmic surgeries. Dr. Irina Vasilcenko performed oral and maxillofacial surgery.

In the first stage of the surgical treatment, a revision of the wound was performed by the department of ophthalmology. In the beginning, a dissection of the conjunctival tissue along the 9 to 3 o’clock positions in the vicinity of the affected site was made (**Fig. 1C**). Once the bulging defect was approached and initiated, the tissues of the crystalline lens were discovered to form the majority of the defect's bulk volume (**Fig. 1D**). The surgical team then proceeded to extract the dislocated crystalline lens tissues while simultaneously reducing the volume of the defect. The scleral defect was found and closed with seven interrupted sutures (**Fig. 1E**). Finally, the prolapsed scleroconjunctival tissues were repositioned, and the wounds were closed with sutures (**Fig. 1F**).

For the second stage of treatment, the patient was transferred to the Department of Maxillofacial Surgery. The infraorbital margin of the left eye was accessed via a subciliary incision. The left zygomatic bone was repositioned using a Limberg hook, and osteosynthetic fixation was performed with two microplates and eight microscrews. A defect in the inferior orbital wall was repaired with a titanium mesh and two microscrews. Finally, the surgical wound was closed with Novosin 4/0 and Dafilon 5/0 sutures.

For the third and final stage of treatment, the patient returned to the Department of Ophthalmology a month later (March 24, 2024). The patient presented with aphakia and total hemophthalmos in the left eye. An iridodialysis was found along the 9 to 10 o’clock position, with a defect of the iris at the 11 o’clock position. A 25G pars plana vitrectomy was performed in the left eye to remove blood clots from the anterior chamber, along with the removal of residual fibrotic membranes (**Fig. 1A, B**). After a complete vitrectomy was performed to remove the hemophthalmos (**Fig. 1C**), an Artisan iris-fixated intraocular lens (IOL) was implanted and secured retropupillary via a corneal incision. The corneal wound was closed with a Nylon 10/0 cross suture (**Fig. 1D**).

**Fig. 2:**
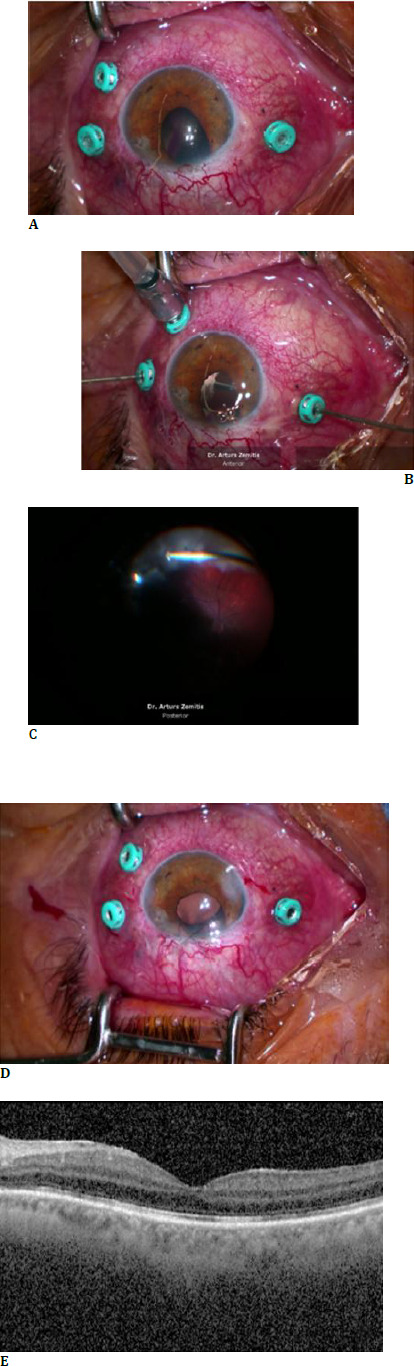
A follow-up surgery on March 24, 2024, involved a 25-G pars plana vitrectomy (**A**). Removal of residual fibrotic membranes (**B**). Complete vitrectomy for hemophthalmos removal (**C**). Artisan iris-fixated intraocular lens (IOL) implantation and retropupillary securing via a corneal incision and closing the scleral wound with a cross suture (**D**). At the latest examination on May 3, 2024, the patient presented with a mild eye injection and a corneoscleral cross suture located at the 12 o’clock position (**E**). The optical coherence tomography (OCT) performed demonstrated a preserved macular structure (**F**)

## Results

Immediately after the first stage of the treatment, the patient presented with a BCVA of 20/400 in the left eye. One month following the eye trauma, during the third stage of treatment involving intraocular lens (IOL) implantation, the patient’s BCVA in the left eye improved to 20/100.

Upon a follow-up visit two weeks later, the best-corrected visual acuity (BCVA) in the left eye was found to be 20/70. Biomicroscopic eye examination presented light conjunctival injection, the intraocular lens position was stable, and the cross suture was seen in the upper quadrant at the 12 o'clock position. Optical coherence tomography (OCT) demonstrated a preserved macular structure of the left eye (**Fig. 1E, F**).

## Discussion

Phacocele has a low incidence rate and can be found in up to 13% of all lens luxations. The force of blunt eye globe trauma can cause an indirect rupture in the scleral wall, leading to the crystalline lens passing through into the subconjunctival space.

The most prevalent risk factors include prior eye globe trauma or surgical procedures, such as cataract surgery, vitrectomy, or trabeculectomy. However, in the case we are describing, the patient had no history of previous trauma or eye injuries. In this case, the force of blunt eye trauma alone resulted in crystalline lens luxation into the subconjunctival space [4,5].

Due to the rare incidence of traumatic phacocele, there is a lack of available literature and documented cases. In the cases described by Neupane S and Vodapalli H, the phacocele was most commonly located in the superonasal quadrants, towards the 12 o’clock position [6,7]. Bhattacharjee K has, however, described a superotemporal position of crystalline lens dislocation [8], suggesting that the predominant luxation of the phacocele can be either the upper nasal or temporal position, which was also observed in this particular case.

The primary treatment for the phacocele is surgical intervention, which involves exploring the wound, dislocating the lens, and performing a vitrectomy. In this case, surgical intervention was promptly carried out within a day of the report without any delay. The surgery was performed in three steps, including a maxillofacial procedure. To prevent infection of the wound or endophthalmitis, the insertion of the intraocular lens (IOL) was deferred and successfully performed one month after the initial surgery.

Postoperative visual acuity can vary due to several factors, including the size of the scleral wound, lens insertion, vitreous haemorrhage, and retinal breaks. Based on the Bhattacharjee K case series, the BCVA can range from hand motion to complete visual recovery [8]. Following Kuhn’s Ophthalmic Trauma Score (OTS), as the patient’s best-corrected visual acuity (BCVA) was hand motion at the time of presentation, the raw sum score corresponds to 70 points, with no other initial visual acuity factor impacting the score. The total number of points corresponds to an OTS score of 3, with a probability of achieving a BCVA of 20/200 to 20/50 in 28% of cases and a BCVA of 20/40 or better in 44% of cases, six months after the incident [9]. In the case we have described, the patient’s BCVA demonstrated significant improvement, reaching 20/70. However, the presence of post-vitrectomy complications and corneoscleral sutures may potentially lead to a decline in vision. To minimise suture-related complications such as astigmatism, it is preferable to remove the sutures at least 8 weeks postoperatively [10].

## Conclusion

Phacocele is defined as the displacement of the crystalline lens into the subconjunctival space and is a rare complication that may arise from blunt eye trauma. Due to its infrequency, there is limited literature and documented cases available. However, existing cases confirm its occurrence. The primary treatment approach typically involves immediate eye surgery, including wound revision and lens extraction. As demonstrated in this case, the patient exhibited significant improvement in best-corrected visual acuity (BCVA) following surgical interventions in the eye.
